# Use of Oxfendazole to Control Porcine Cysticercosis in a High-Endemic Area of Mozambique

**DOI:** 10.1371/journal.pntd.0001651

**Published:** 2012-05-29

**Authors:** Alberto Pondja, Luís Neves, James Mlangwa, Sónia Afonso, José Fafetine, Arve Lee Willingham, Stig Milan Thamsborg, Maria Vang Johansen

**Affiliations:** 1 Faculty of Veterinary Medicine, Eduardo Mondlane University, Maputo, Mozambique; 2 Biotechnology Centre, Eduardo Mondlane University, Maputo, Mozambique; 3 Faculty of Veterinary Medicine, Sokoine University of Agriculture, Morogoro, Tanzania; 4 Department of Veterinary Disease Biology, Faculty of Health and Medical Sciences, University of Copenhagen, Copenhagen, Denmark; Universidad Nacional Autónoma de México, Mexico

## Abstract

A randomized controlled field trial to evaluate the effectiveness of a single oral dose of 30 mg/kg of oxfendazole (OFZ) treatment for control of porcine cysticercosis was conducted in 4 rural villages of Angónia district, north-western Mozambique. Two hundred and sixteen piglets aged 4 months were selected and assigned randomly to OFZ treatment or control groups. Fifty-four piglets were treated at 4 months of age (T1), while another 54 piglets were treated at 9 months of age (T2) and these were matched with 108 control pigs from the same litters and raised under the same conditions. Baseline data were collected on the prevalence of porcine cysticercosis using antigen ELISA (Ag-ELISA), as well as knowledge and practices related to *Taenia solium* transmission based on questionnaire interviews and observations. All animals were followed and re-tested for porcine cysticercosis by Ag-ELISA at 9 and 12 months of age when the study was terminated. Overall prevalence at baseline was 5.1% with no significant difference between groups. At the end of the study, 66.7% of the controls were found positive, whereas 21.4% of the T1 and 9.1% of the T2 pigs were positive, respectively. Incidence rates of porcine cysticercosis were lower in treated pigs as compared to controls. Necropsy of 30 randomly selected animals revealed that viable cysts were present in none (0/8) of T2 pigs, 12.5% (1/8) of T1 pigs and 42.8% (6/14) of control pigs. There was a significant reduction in the risk of *T. solium* cysticercosis if pigs were treated with OFZ either at 4 months (OR = 0.14; 95% CI: 0.05–0.36) or at 9 months of age (OR = 0.05; 95% CI: 0.02–0.16). Strategic treatment of pigs in endemic areas should be further explored as a means to control *T. solium* cysticercosis/taeniosis.

## Author Summary

Porcine cysticercosis is an infection of pigs caused by the larval stage of *Taenia solium*, a tapeworm that causes taeniosis in humans. The disease is very common in developing countries where it is a serious public health risk and causes significant economic losses in pig production. Many control strategies in developing countries have been of limited impact mainly due to poor socioeconomic and sanitary conditions. An effective treatment of infected pigs using inexpensive drugs may have potential as a long term control tool. We performed a randomized controlled trial to evaluate the effectiveness of oxfendazole treatment for control of porcine cysticercosis. We evaluated the prevalence and incidence of the disease in groups of pigs treated at 4 and 9 months of age and untreated pigs. We found that the prevalence and incidence of the disease in treated pigs was significantly lower than in untreated pigs. We conclude that treatment of pigs with oxfendazole in the last part of the fattening period is cost-effective in controlling porcine cysticercosis in endemic low-income areas but should be integrated with other control measures.

## Introduction

*Taenia solium* is the etiologic agent of cysticercosis, an important zoonotic infection involving humans and pigs. The life cycle of this parasite includes pigs as the normal intermediate hosts, harbouring the larval cysts in many parts of the body causing cysticercosis, and humans as definitive hosts, harbouring the adult tapeworm in the intestines causing a condition known as taeniosis. Humans are accidental hosts of cysticerci after ingestion of *T. solium* eggs from the environment and develop the cysts in their tissues and organs, with the central nervous system (CNS) being a common site of cyst location resulting in neurocysticercosis [1], [2]. Cysticercosis in pigs is endemic in many developing countries of Latin America [3], [4], Africa [5], and Asia [6], where it causes important economic losses resulting from condemnation of infected pork [7], [8]. The disease has been declared preventable and potentially eradicable [9], but in many developing countries it is still a major constraint in pig production mainly due to lack of awareness about its extent, poor socioeconomic conditions and the absence of suitable diagnostic tools and control strategies [10]–[13].

Currently, the diagnosis of porcine cysticercosis in live animals is based on lingual examination that is sensitive only in detecting moderate to heavy infections [14]. Reliable serological tests based on detection of specific antibody and antigen have been developed and proved very useful in confirming diagnosis [15], [16]. Among them the Ag-ELISA has been reported to have high specificity (86.7%) and sensitivity (94.7%), even detecting circulating antigens in pigs harbouring one single *T. solium* cyst [15], [17] or detecting circulating antigens as early as two to six weeks after infection [17]. However, the detection of circulating antigens technique is unable to distinguish *T. solium* from *T. hydatigena* cysticerci, and where the later parasite is highly prevalent the method may be of limited use [18].

Control measures such as improved animal husbandry practices, efficient meat inspection procedures, and health education about hygiene and sanitation have been of limited impact in developing countries where pigs are mainly raised by poor smallholder farmers and marketing of pork is not controlled [19]. However, control of cysticercosis should be possible by eliminating the infection from either pigs or humans, or both for an extended period. Since the pig constitutes a vital link in the transmission cycle of *T. solium* [20], [21], an effective treatment of infected pigs should interrupt the transmission cycle. Oxfendazole (methyl 5[6]-phenylsulfinyl-2-benzimidazolecarbamate) (OFZ), a benzimidazole anthelmintic commonly used in cattle and small ruminants for treatment and control of gastro-intestinal roundworms, lungworms and certain tapeworms, has been shown to kill all viable *T. solium* cysticerci in muscles but ineffective against brain cysts in infected pigs [22]–[25]. Pigs treated with OFZ were reported to be refractory to re-infection even in the event of ongoing exposure to *T. solium* eggs [25]. More importantly, carcasses from treated pigs were reported to have a normal appearance suitable for human consumption after 3–6 months depending on intensity of infection [23], [24]. Surveillance for detection of infected pigs followed by treatment with OFZ could reduce the flow of contaminated pork into the market [22], [23]. Mass porcine chemotherapy with OFZ could, therefore, also be a useful strategy to control *T. solium*, by providing health as well as economic benefits for rural poor smallholder communities. However, most studies that have addressed the use of OFZ against porcine cysticercosis thus far have mainly focused on efficacy of the drug as opposed to its effectiveness. Therefore, the present study aimed to evaluate the effectiveness of a single oral dose of 30 mg/kg of OFZ against *T. solium* cysticercosis in pigs reared in smallholder farming systems in a highly endemic area.

## Materials and Methods

### Study area

The study was conducted in Angónia district located in north-western Mozambique between latitude 14.27°S and 15.28°S, and longitude 33.59°E and 34.38°E. The district is characterized by a humid climate with a rainy season extending from November to mid-March and the dry period from April to October. Four villages were selected randomly from a group of 11 villages within the district with a known high prevalence of porcine cysticercosis [26]. A preliminary visit was made to the selected villages to evaluate villagers' willingness to collaborate in a longitudinal study for control of porcine cysticercosis.

### Experimental study design

A randomized field study with a control group was conducted, between August 2008 and May 2009, to evaluate the effectiveness of a single oral dose of 30 mg/kg of OFZ treatment against *T. solium* cysticercosis by comparing the prevalence and incidence of porcine cysticercosis between treatment and control groups. Fifty-four pig litters from same number of households, comprising a total of 216 piglets aged 4 months were selected for the study and followed to the age of 12 months. All piglets were identified and ear-tagged with consecutive numbers and each litter in a household was divided into three groups by randomization. The Group 1 piglets (n = 54) were treated with OFZ at 4 months of age (T1), Group 2 (n = 54) was treated at 9 months of age (T2) while Group 3 (n = 108) served as non-treated controls (C). At the end of the trial, a total of 30 randomly selected pigs (8 from each of the OFZ-treatment groups and 14 from the control group) were purchased from villagers, slaughtered locally and dissected for assessment of *T. solium* cysticerci.

### Blood collection

Blood was collected from all animals in both treatment and control groups in three sampling rounds (4, 9 and 12 months of age), and information regarding sampling date, household, village, sex and age was recorded. Blood samples were obtained from the cranial vena cava into plain vacutainers tubes and allowed to clot at 4°C. Serum was obtained by centrifugation, dispensed into 2 ml aliquots, stored in labelled vials and kept at −20°C until use.

### Oxfendazole treatment of experimental animals

The animals of the two OFZ-treatment groups were first weighed and later given orally a single dose of 30 mg/kg OFZ (Oxfen-C, Lot 800869, Bayer, Isando, South Africa) as a suspension (concentration of 9.06%), while the control group did not receive any treatment. The drug was administered through a dispensing tube attached to a 15 ml drench gun. The pig was firmly restrained and a pig snare was used to stabilize the head. The end of the dispensing tube of the drench gun was passed gently, but firmly, over the back of the tongue to allow the pig to swallow the dispensed suspension and ensure the complete delivery of the drug.

### Serological detection of circulating antingens of *T. solium* cysticerci (Ag-ELISA)

The Ag-ELISA was performed as described by Brandt and others [27] and modified by Dorny and others [15]. Briefly, the serum samples were pre-treated using trichloroacetic acid (TCA) and used in ELISA at a final dilution of 1/4. Two monoclonal antibodies (MoAb) used in a sandwich ELISA were B158C11A10 (Lot K, ITM, Antwerp, Belgium) diluted at 5 µg/ml in carbonate buffer (0.06M/pH 9.6) for coating and a biotinylated MoAb B60H8A4 (Lot 28, ITM, Antwerp, Belgium) diluted at 1.25 µg/ml in phosphate buffered saline-Tween 20 (PBS-T20)+1% new born calf serum (NBCS) as detector antibody. The incubation was carried out at 37°C on a shaker for 30 min for the coating of the first MoAb and for 15 min for all subsequent steps. The substrate solution consisting of ortho phenylenediamine (OPD) and H_2_O_2_ was added and incubated without shaking at 30°C for 15 min. To stop the reaction, 50 µl of H_2_SO_4_ (4N) was added to each well. The plates were read using an ELISA reader at 492 nm. Sera from two known positive pigs (confirmed at slaughter) were used as positive control. To determine the cut-off, the optical density (OD) of each serum sample was compared with a series of 8 reference negative serum samples at a probability level of 0.1% using a modified Student's *t*-test [28].

### Carcass dissection for assessment of porcine cysticercosis

Carcass dissection was performed as described by Phiri *et al* [14] with slight modifications. Briefly, skeletal muscle groups were excised from the left half carcasses together with the complete heart, tongue, head and neck muscles, psoas muscles, diaphragm, lungs, kidneys, liver and brains. Dissection was done in such a way that all fully developed cysts could be revealed (i.e. each slice was about 0.5 cm thick). Animals were considered infected if viable cysts were found in the carcass.

### Data analysis

Data were entered and analysed using STATA version 9.1 (Stata Corporation, College Station, TX, USA). Prevalence estimates were calculated for pigs that were bled in each sampling round. Incidence was estimated as the number of new cases occurred per unit of animal time at risk, during a period between two consecutive sampling rounds. Confidence intervals were calculated for prevalence and incidence of porcine cysticercosis in both treatment and control groups. Chi-square test and statistical comparison of rates were used to compare the prevalence and incidence, respectively. Logistic regression models were fitted to examine the role of factors potentially associated to prevalence of porcine cysticercosis. Examined factors included treatment time, sex of the pig, pig husbandry practices and village. The statistical significance level was set at 5%.

### Ethics statement

The study was conducted with ethical approval from the scientific board of the Veterinary Faculty, Eduardo Mondlane University. All animals were handled in strict accordance with good animal practice as defined by the OIE's Terrestrial Animal Health Code for the use of animals in research and education. Study permissions were obtained from the Livestock National Directorate, village leaders and pig owners. Due to high level of illiteracy among villagers, an oral consent was obtained from pig farmers in the presence of a witness, who signed on their behalf. At the end of the trial all animals were treated with OFZ, and pig farmers were informed not to slaughter their animals before 4 weeks after the treatment.

## Results

### Experimental animals and sampling

All examined pigs (n = 216) were of the local breed (Landim), mostly males (55%) and the majority (93.1%) were deliberately left to roam freely. A total of 570 samples were collected along three sampling rounds from 216 pigs. From these animals, 32 (14.8%) were sampled once, 184 (85.2%) were sampled twice, and 170 (78.7%) were sampled three times. Altogether 46 animals were lost to follow-up, out of which 24 (22.2%) were from the control group, 12 (22.2%) from T1 group and 10 (18.5%) were from T2 group. The main reasons for losses of animals to follow-up were death, sale or refusals to allow sampling due to absence of the head of the household. Overall baseline prevalence at 4 months of age was 5.1% (95% CI = 2.6%–8.9%) and did not differ significantly (p>0.05) among comparison groups.

### Measurement of treatment effectiveness

#### Prevalence

Comparison of prevalence between treatment and control groups showed similar results at baseline but lower in both treatment groups at the end of the study (Table 1). A significant increase (p<0.001) in the prevalence of porcine cysticercosis was observed in the control group during all the study period. A similar increasing trend was also observed in T1 pigs although the prevalence was significantly lower (p<0.001) compared to the control group at 12 months of age. In T2 pigs, the prevalence of porcine cysticercosis increased significantly (p<0.001) from 4 to 9 months of age, but was significantly reduced (p<0.01) at 12 months of age. Although the prevalence was higher in the T1 group than in the T2 group at month 12 of age, the difference was not statistically significant (p = 0.11).

**Table 1:** Prevalence of porcine cysticercosis.

Age	Control group		T1 group		T2 group	
	Number tested	Prevalence (%)	Number tested	Prevalence (%)	Number tested	Prevalence (%)
4 months	108	5.6	54	5.5	54	3.7
9 months	90	33.3	44	13.6	50	36.0
12 months	84	66.7	42	21.4	44	9.1

#### Incidence

At the baseline, a total of 205 pigs from all groups were negative by Ag-ELISA, thus being at risk of infection Comparison of incidence between treatment and control groups showed lower incidence rates (p<0.05) in both treatment groups during the follow-up period (Table 2). In addition, all infected pigs at the time of treatment were found negative in the subsequent sampling round. However, apart from the new cases observed after treatment and losses to follow-up, 33.3% (n = 1) of infected pigs treated at 4 months of age were found re-infected at 12 months of age, whereas all (n = 18) infected pigs treated at 9 months of age were found free of *T. solium* circulating antigens at the age of 12 months.

**Table 2:** Incidence rates of porcine cysticercosis.

Period	Number of cases per 100 pigs-month		
	Control group	T1 group	T2 group
1 (between 1^st^ and 2^nd^ sampling)	2.2	1.1	2.9
2 (between 2^nd^ and 3^rd^ sampling)	11.5	2.1	1.6

#### Cyst assessment

Carcass dissection of the 30 randomly selected pigs at the end of the study revealed that 12.5% (1/8) of T1 pigs and 42.8% (6/14) of control pigs had viable cysts in different organs and muscles, whereas this was the case in none of the T2 pigs (Table 3). Cysts were frequently localized in group muscles (82.1%), heart (5.6%), tongue (4.8%), diaphragm (4.5%) and brain (2.8%). However, brain cysts were only observed in some (n = 3) control pigs. The number of cysts ranged between 17 and 113 with an average of 50 cysts per pig. All pigs with *T. solium* cysticerci at necropsy were also found positive by Ag-ELISA.

**Table 3:** Number of pigs with *Taenia solium* cysts at necropsy.

Findings at necropsy	Control group	T1 group	T2 group
One or more viable cysts	6	1	0
No cysts	8	7	8
Total	14	8	8

#### Multivariate analysis

Multivariate logistic regression analysis of the effect of treatment (at the end of experiment) adjusted for sex of the pig, husbandry system and village (Table 4) revealed a significant (p<0.001) decrease in the risk for porcine cysticercosis infection at the age of 12 months if pigs were treated with OFZ either at 4 (OR = 0.14; 95% CI: 0.05–0.36) or at 9 months of age (OR = 0.05; 95% CI: 0.02–0.16).

**Table 4:** Multiple logistic regression analysis for factors associated with prevalent cases of porcine cysticercosis.

Factor		Odds Ratio	95% CI	p-value
Treatment group	Control	1		
	OFZ-T1	0.14	0.05–0.36	<0.001
	OFZ-T2	0.05	0.02–0.16	<0.001
Sex	Female	1		
	Male	1.02	0.47–2.22	0.95
Free range	No	1		
	Yes	1.76	0.38–8.20	0.47
Village	Camuetsa	1		
	Campessa	1.12	0.34–3.68	0.85
	Ndaula	1.09	0.40–2.95	0.87
	Lilanga	1.06	0.23–4.81	0.94

## Discussion

This study in Angónia district has evaluated the effectiveness of OFZ treatment in pigs, as a strategy to control *T. solium* cysticercosis in a highly endemic area in which pigs are constantly exposed to the parasite. A substantial benefit of treating pigs with OFZ using the single oral dose of 30 mg/kg body weight was clearly demonstrated, since the prevalence and incidence in groups of treated pigs was significantly lower compared to the group of untreated pigs. All pigs that were infected at the time of treatment with OFZ were found negative in the subsequent sampling round (5 and 3 months later for T1 and T2, respectively). Interestingly, this result was observed in animals raised under constant exposure to *T. solium* eggs [26] but were in keeping with previous studies conducted under controlled settings reporting a clear effect of OFZ in killing cysts when given as a single dose of 30 mg/kg [22]–[24], [29].

A significant number of negative control pigs (51/79) got infected whereas 1 of the 2 infected pigs treated at 4 months and none of the 18 infected pigs treated at 9 months were found re-infected at the end of the study. The latter result is very convincing of induction of a high level protective immunity whereas the former may raise some speculations. One could argue that the result may be explained by differences in individual immune responsiveness or even exposure, as animals become susceptible to new infections after treatment. Also, it can be speculated that there were false serological results or even cross reactions with *T. hydatigena* infection, though this is unlikely as none of the 51 pigs found infected in control group had fluctuating positivity and all pigs (7/30) that were found positive at necropsy had no *T. hydatigena* cysticerci. Nevertheless, in accordance with our findings, naturally infected pigs treated with OFZ under field conditions were not re-infected for at least 3 months. These results support conclusions from previous studies [23], [25] and speculations that treated pigs remain immune to re-infection for at least 12 weeks [24].

However, although the effectiveness of OFZ can be regarded as good, the prevalence and incidence of *T. solium* cysticercosis in T1 pigs increased during the study period. The observed increase after treatment is most likely related to new infections in animals being fully susceptible at the time of treatment. Indeed, nearly all of the T1 pigs (51/54) were negative by Ag-ELISA at the time of treatment, thus being at risk of infection. On the other hand, the prevalence and incidence of *T. solium* cysticercosis in T2 pigs reduced significantly from treatment at 9 months to 12 months of age. This strategy, although also involving possible treatment of uninfected pigs, showed better results and the potential to be used as a control strategy for *T. solium* cysticercosis in our settings considering the lifespan of pigs is usually less than one year. Our findings are in line with an earlier field study that showed a clear effect of mass treatment with OFZ at 30 mg/kg body weight in decreasing the prevalence and incidence of porcine cysticercosis, however that study had utilised an antibody-detection method which measures exposure and not necessarily active infection [30].

The significant decrease in the risk of porcine cysticercosis infection at the age of 12 months observed in this study if pigs were treated with OFZ, either at 4 or 9 months of age, was observed in a context of poor living conditions in the study area, where most pigs were left to roam freely and no other control interventions were implemented. This finding, and the low cost of the drug (approximately 0.018 USD/dose), corroborates previous conclusions considering OFZ as a potential effective control tool for porcine cysticercosis in resource-poor endemic areas [22]–[24], [29]. However, it should be borne in mind that many strategies to control *T. solium* cysticercosis in developing countries have shown promising results [7], [13], [21], [31], [32] but none has fully succeeded up to date, mainly due to poor socioeconomic and sanitary conditions [7], [13]. Control measures targeting pigs alone would be ineffective as they cannot prevent the spread of cysticercosis in humans and pigs [21], [33], [34], thus there is a need for integration with other *T. solium* control strategies in the long term [34]–[36]. Moreover, inexpensive and reliable diagnostic tests in live animals are needed for monitoring the effect of interventions in endemic countries. The Ag-ELISA used in this study, though considered very sensitive [37] may have drawbacks in monitoring the effectiveness of treatment in pigs as cysticercal antigen levels take some time to disappear from circulation after treatment depending on the intensity of infection [24]. Furthermore, the technique may not detect brain cysts [24], [25], though in our study brain cysts were only found in some of the control pigs, and currently it does not allow differentiating *T. solium* from *T. hydatigena* cysticerci [18].

The results presented in this study showed that OFZ treatment in the last part of the pigs' fattening period is effective to control porcine cysticercosis but is not a stand-alone approach because in high endemic areas a certain number of animals will inevitably get infected after treatment and before slaughter. Although effective, its strategic use as a control tool in *T. solium* endemic areas should be further explored, particularly with regard to availability, formulation, regimen of administration, safety and marketing of pigs. Despite these concerns and considering that any strategy to control *T. solium* by targeting pigs has a potential to provide economic incentives to poor smallholder pig farmers, treatment of pigs with OFZ, if integrated with other control measures such as treatment of human tapeworm carriers, ending open human defecation, education, and community-based inspection and sales restrictions, should be considered an important, cost-effective measure to reduce the transmission of *T. solium* infections in endemic low-income areas.
